# Is Danggui Safe to be Taken by Breast Cancer Patients?—A Skepticism Finally Answered by Comprehensive Preclinical Evidence

**DOI:** 10.3389/fphar.2019.00706

**Published:** 2019-06-25

**Authors:** Grace Gar-Lee Yue, Lok-Sze Wong, Hoi-Wing Leung, Si Gao, Julia Yuen-Shan Tsang, Zhi-Xiu Lin, Bonita Ka-Bo Law, Gary Man-Kit Tse, Clara Bik-San Lau

**Affiliations:** ^1^Institute of Chinese Medicine, The Chinese University of Hong Kong, Shatin, New Territories, Hong Kong; ^2^State Key Laboratory of Research on Bioactivities and Clinical Applications of Medicinal Plants, The Chinese University of Hong Kong, Shatin, New Territories, Hong Kong; ^3^Department of Anatomical and Cellular Pathology, The Chinese University of Hong Kong, Shatin, New Territories, Hong Kong; ^4^School of Chinese Medicine, The Chinese University of Hong Kong, Shatin, Hong Kong; ^5^Union Hospital, Shatin, Hong Kong

**Keywords:** breast cancer, *Angelica sinensis*, safety, estrogenic herbs, primary breast cancer cells, Chinese herbal medicines

## Abstract

*Angelica sinensis* (AS, Danggui) has long been regarded to stimulate breast cancer growth; hence, the use of AS in breast cancer patients remains a major concern for both patients and practitioners. Since safety studies of herbs would be unethical to carry out in patients, the present study aimed to investigate the potential unsafe effects of AS in a systematic pre-clinical approach. Human breast cancer cells, breast orthotopic tumor-bearing mouse models, as well as primary breast cancer cells from patients’ tumors were used to evaluate the effect of AS hot water extract on the progression of breast tumors and/or growth of breast cancer cells. We showed that AS is not that stimulatory in breast cancer both *in vitro* and *in vivo*, though AS should still be used with caution in estrogen receptor-positive breast cancer patients. This novel approach of applying breast cancer cell lines, xenograft, and syngeneic tumors models, as well as primary breast cancer cells from patients’ tumors in Chinese medicines safety evaluation was proven feasible. Our finding is important information for patients, Chinese medicine practitioners, and clinicians on the safety use of AS in breast cancer, which will affect future clinical practice.

## Introduction

Breast cancer is the most frequently diagnosed cancer among women worldwide with an estimated 1.67 million new cancer cases diagnosed in 2012 (25% of all cancers) (Ferlay et al., 2018). In China, estimated incidence was around 190,000 in 2012. In Hong Kong, the incidence rate of breast cancer is the highest in female, accounting for 26.1% of total cancer cases in 2015 (Hong Kong Hospital Authority, 2018). The use of complementary and alternative medicine (CAM) as an adjuvant to cancer treatment is more frequent among breast cancer patients all over the world (DiGianni et al., 2002; Boon et al., 2007; Davis et al., 2010; Wong et al., 2014; Dietz et al., 2016). A survey on CAM use in the Scottish breast cancer population also showed that 33.1% of respondents reported using CAM, including dietary supplements (McLay et al., 2012). Chinese herbal medicine (CHM) is a popular form of CAM among Chinese population (Lam et al., 2009; Wong et al., 2010; Lin and Chiu, 2011; Liu et al., 2012; Fan et al., 2014).

A Cochrane intervention review reported that Ginseng Radix, Angelicae Sinensis Radix, and Astragali Radix were prescribed to breast cancer patients for managing the side effects of chemotherapy in several clinical trials (Zhang et al., 2007). Besides, Chinese herbal products containing Ginseng Radix or *Angelicae Sinensis* Radix were the most frequently prescribed for breast cancer patients in Taiwan (Lai et al., 2012). The common pharmacological characteristic of these herbal medicines is their estrogenic effects (Amato et al., 2002; Lee et al., 2003; Gao et al., 2007). Botanicals containing estrogenic compounds were suggested to have potential benefits for women’s health, such as alleviate the symptoms of menopause (Piersen, 2003). However, dietary phytoestrogens (e.g., soy) may also have promoting effects on tumor recurrence (Roberts, 2010). The potential risks of estrogenic dietary supplements consumption by breast cancer patients or cancer survivors were aroused for over a decade (Piersen, 2003; Rice and Whitehead, 2006). There are CHMs commonly prescribed for gynecological complaints being shown to contain phenolic phytoestrogens (He et al., 2002; Piersen, 2003).

**Figure 1:**
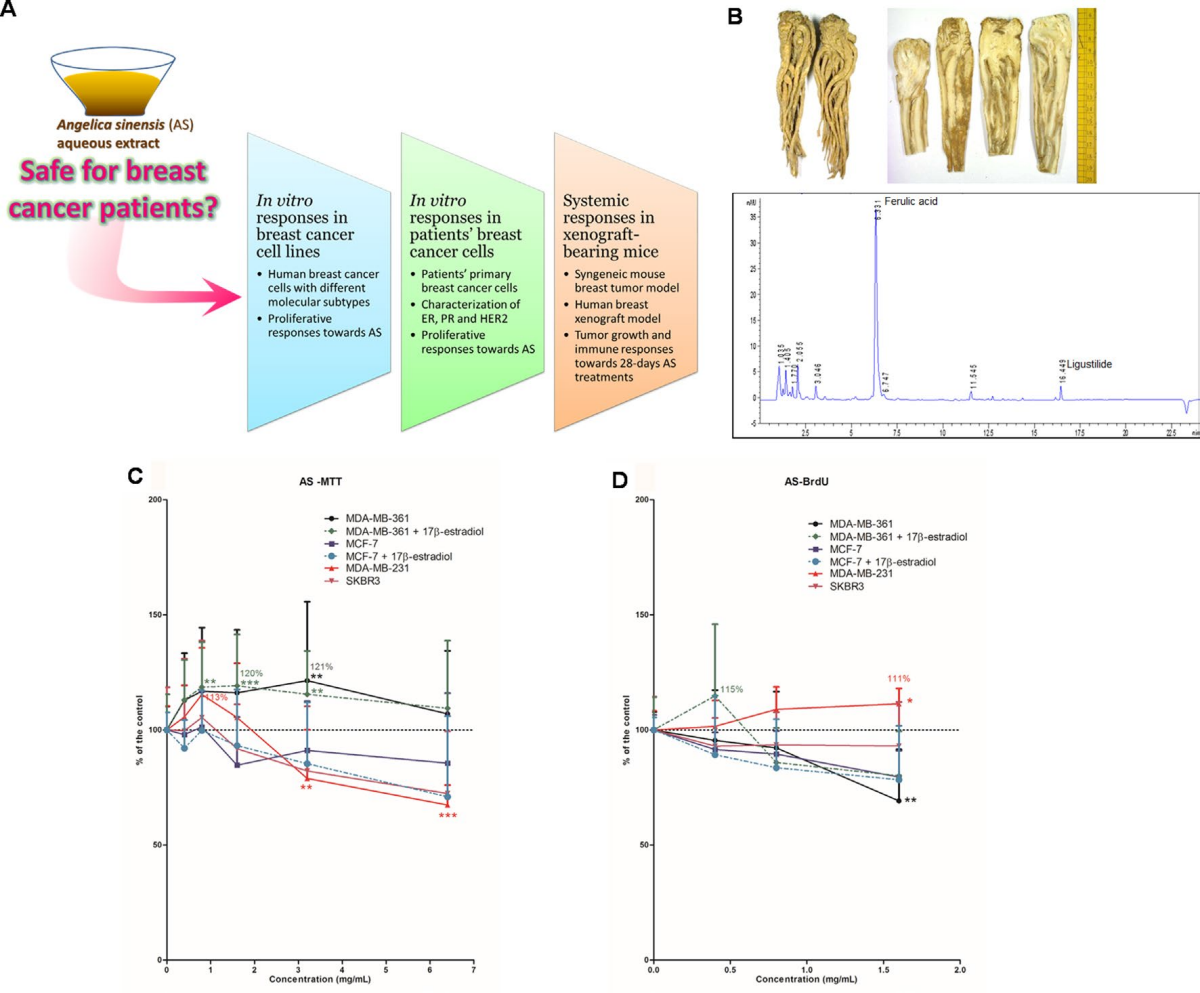
Study flow and chemistry of *Angelicae Sinensis* Radix. **(A)** Schematic diagram showing the experimental flow of the present pre-clinical study. **(B)** Dried herb (whole piece and slices) of *Angelicae Sinensis* Radix (AS) and the representative UPLC chromatogram of AS extract. **(C, D)** Effects of AS aqueous extract on human breast cancer cells. **(C)** Cell viability and **(D)** cell proliferation in MDA-MB-361, MCF-7, MDA-MB-231, and SKBR3 cells. Cells were treated with various concentrations of AS extract **(A)** 0.4–6.4 mg/ml for cell viability assay; **(B)** 0.4–1.6 mg/ml for proliferation assay) alone or in combination with 0.1 µM of 17β-estradiol for 48 h. Data were expressed as the mean percentage of the untreated control (in **C** and **D**: three independent experiments with five replicates each; in **B**: two independent experiments with three replicates each). Statistical differences were determined by one-way ANOVA, followed by Dunnett Test, with *p < 0.05, **p < 0.01, ***p < 0.001 against untreated control.

The safety use of estrogenic CHMs, such as *Angelica sinensis* (Oliv.) Diels, AS, in estrogen-dependent cancer patients remains confusing for years, especially for the Chinese medicine practitioners and some CAM users in Western countries, and it has seldom been reviewed or investigated. A previous study has evaluated the effects of four selected herbs commonly used in menopause on the growth of breast cancer cells and has demonstrated that the ethanolic extract of Danggui (*Angelicae Sinensis* Radix, dried root of AS) stimulated MCF-7 cells growth *in vitro* (Amato et al., 2002). Our previous study also showed that AS water extract stimulated the growth of MCF-7 cells, possibly dependent of weak estrogen-agonistic activity, and augmented the BT-20 cell proliferation independent of estrogen receptor (ER)-mediated pathway (Lau et al., 2005). Another study showed the increased proliferation of HeLa cells by AS water extract (Zhu et al., 2007). The active compound from AS, ferulic acid, has also been reported to cause breast cancer cell proliferation by up-regulation of HER2 and ERα expressions (Chang et al., 2006). Nevertheless, the effects of AS in breast cancer *in vivo* models have seldom been reported. Up till now, there are in fact no definite answers as to whether AS will promote tumor growth in breast tumor-bearing animals or in human. However, cancer patients after chemotherapy will usually be prescribed with tonifying and/or invigorating herbs (e.g., AS) by Chinese medicine practitioners. In addition, tonifying herbs such as AS may also be included in Chinese cuisine dishes. Some of the tonifying herbs have been shown to have estrogenic effects as mentioned. The consumption of these herbs by breast cancer patients is therefore not uncommon but the safety of consuming these herbs by breast cancer patients is still unclear. Clinical study on the effects of such CHM in breast cancer patients or survivors will be ideal; nevertheless, it is not ethical nor feasible due to the potential harmful outcomes. Hence, a systematic study approach in tackling this issue is highly warranted. In this study, we managed to design and implement a series of pre-clinical experiments/assessments, in which human breast cancer cell lines, primary human breast cancer cells isolated from informed and written consented patients’ tissues, and breast tumor-bearing mice models were adopted to evaluate the potentially unsafe effects (proliferation of cancer cells or promotion of tumor growth) caused by AS treatment (**Figure 1A**).

Breast cancer is a heterogeneous disease and can be classified into different subtypes, which have different prognosis and treatment responses (Holliday and Speirs, 2011). Expressions of the ER, progesterone receptor (PR), and/or human epidermal growth factor receptor 2 (HER2) have long been used for breast cancer classification (Hollestelle et al., 2010). As ER and HER2 are the therapeutic targets, those receptor-positive breast cancers can be treated by hormone therapy; while those triple receptor-negative breast cancers may associate with poor prognosis (Holliday and Speirs, 2011). Hence, human breast cancer cell lines with different immunoprofiles were used to evaluate their proliferative responses after AS treatment *in vitro*. While the most sensitive breast cancer cell line was selected for subsequent *in vivo* xenografts model in athymic nude mice. In the meantime, it would be desirable to include primary cells derived from tumors with different molecular subtypes to generalize the findings of AS effect on the breast cancer cell growth. Here, we successfully isolated the primary breast cancer cells from patients’ tissues and the proliferative responses of these cells towards AS treatments were demonstrated for the first time, which provided more clinically relevant information than previous studies using breast cancer cell lines.

Apart from evaluating the effects of AS treatments, i.e., oral administration of AS aqueous extract (abbreviated as AS extract) at Chinese pharmacopoeia recommended dosages, on the growth of orthotopic human breast xenografts in nude mice, as well as the systemic influences of AS treatments (with or without chemotherapeutic) were also examined in syngeneic breast tumor-bearing mice models. Many cancer patients who underwent chemotherapies were categorized to have blood-deficiency syndrome according to Chinese medicine theory (Chien et al., 2013) and prescribed with blood-tonic CHM (such as AS). A disease (breast tumor-bearing)–syndrome (blood deficiency) mouse model was established in order to mimic the clinical scenario that breast cancer patients took AS extract to replenish the body after chemotherapies. The blood deficiency mouse model was induced by receiving cyclophosphamide treatment (Li et al., 2011; Gu et al., 2013; Zhang et al., 2014)and the potential detrimental effects of AS treatments were fully revealed in these mice models.

## Materials and Methods

All methods were carried out in accordance with relevant guidelines and regulations from the Chinese University of Hong Kong.

### Herbal Materials and Extraction

A single lot of *Angelicae Sinensis* Radix was purchased from a renowned supplier in Hong Kong and stored in our temperature- and humidity-controlled store room. Both morphological and chemical authentications of the raw herbs were performed in accordance with the Chinese Pharmacopoeia 2015 (Chinese Pharmacopoeia Commission, 2015). Authenticated voucher specimens were deposited in the museum of the Institute of Chinese Medicine, CUHK. The chemical profiles of raw herbs were compared qualitatively using thin layer chromatography (TLC) with the reference herb and reference chemical markers provided by National Institute for the Control of Pharmaceutical and Biological Products. The dried herb was extracted under reflux using water for 1 h and the extraction was repeated once. Following filtration, the crude aqueous extract was centrifuged to remove undissolved particles. The extract was then freeze-dried into powder form. The dried aqueous extract was stored in desiccators at room temperature and avoided sunlight. Extract was dissolved in distilled water for animal studies. In cell studies, extract was dissolved in culture media and filtered before use. The aqueous extract of *Angelicae Sinensis* Radix (AS extract) was subjected to standardization in which the chemical profile was registered and representative chemical markers (ferulic acid and ligustilide) were determined quantitatively using UPLC for quality control purposes (**Figure 1B**).

### *In Vitro* Proliferative Assay in Human Breast Cancer Cells

Four human breast cancer cell lines with different immunoprofiles, namely MDA-MB-361 (ER^+^ HER2^+^), MCF-7 (ER^+^ HER2^-^), SK-BR-3 (ER^-^ HER2^+^), and MDA-MB-231 (ER^-^ HER2^-^) were obtained from American Type Culture Collection (ATCC, USA) and were grown in culture flasks in HuMEC Basal Serum-free Medium (Thermo Fisher, USA) in order to prevent the influence of hormones and/or growth factors in regular serum-containing medium. For ER-positive cell lines (MDA-MB-361 and MCF-7), the cells were seeded in 96-well culture plates and incubated with various concentrations of AS extract, 17β-estradiol (0.1 µM, Sigma, USA), or the combination of 17β-estradiol (0.1 µM) and AS extract in medium for 48 h. For ER-negative cell lines (SK-BR-3 and MDA-MB-231), the cells were seeded in 96-well culture plates and treated with various concentrations of AS extract for 48 h. The cell viability and proliferative responses were determined by 3-(4,5-dimethyl-2-thiazolyl)-2,5-diphenyl-2H-tetrazolium bromide (MTT) assay and bromodeoxyuridine (BrdU) cell proliferation enzyme-linked immunosorbent assay (ELISA) (Roche, USA), respectively.

### Isolation of Human Primary Breast Cancer Cells

All experimental protocols were approved by the Joint Chinese University of Hong Kong–New Territories East Cluster Clinical Research Ethics Committee (CREC Ref. No.: 2014.336) and Union Hospital Ethics Committee (EC Ref. No. EC013). Human primary breast cancer cells were isolated from breast cancer patients’ tumor tissues. Informed and written consents have been obtained from the breast cancer patients (aged over 18) of Union Hospital.

Primary breast cells were obtained from both breast cancerous and adjacent normal tissues after surgical treatment and less than 0.5% by volume of tissues were taken for research purpose. A small part of tumor tissues were visually identified, removed by sterile scalpel blade, and put into appropriate cell culture medium, while the main tumor mass was formalin-fixed and submitted to routine clinical process. The collected breast tumor tissues were divided into ER^+^ or ER^-^ subtypes, which were characterized by immunohistochemistry (Tsang et al., 2012). For primary culture, tissues were mechanically dissociated with scissors and the tissue suspension was incubated in medium containing collagenase and hyaluronidase (Yue et al., 2006). Digestion was monitored under an inverted microscope. The cell suspension was washed with phosphate-buffered saline (PBS) to remove the enzyme and then distributed into culture flasks. Cells were grown in Dulbecco's Modified Eagle Medium (DMEM)/F12 supplemented with 10% (v/v) fetal bovine serum (FBS), 1% (v/v) penicillin and streptomycin, hydrocortisone (10 mg/ml), cholera toxin (200 µg/ml), insulin (10 mg/ml), and epidermal growth factor (200 µg/ml). The primary cell culture could be maintained for up to three passages. When the cells grew to 70% confluent in culture flask, the cells were collected for experiments.

### *In Vitro* Proliferative Assay in Human Primary Breast Cancer Cells

In order to confirm the proliferation responses of breast epithelial cells towards AS extract, the grown primary cells were stained with pan-cytokeratin (epithelial cells marker) and vimentin (fibroblasts marker) (Yeh and Mies, 2008; Smart et al., 2013). The numbers of epithelial cells to fibroblasts were assessed in blinded manner and the ratio of the two types of cells was calculated. The epithelial cells enriched breast cancer cell samples undergone further *in vitro* cell assays. The primary breast cancer cells were seeded in 96-well culture plates and incubated with various concentrations of AS extract or 17β-estradiol (0.1 µM) for 48 h. The cell viability and proliferative responses were determined by MTT assay and BrdU cell proliferation ELISA, respectively.

The correlations of epithelial cells with significant proliferative response for 17β-estradiol (E2) and AS extract were analyzed with chi-square test or Fisher’s exact test. Further analysis on the correlation of ER and PR levels with E2 and AS extract responses was performed on the cases with epithelial cell-enriched primary cells (>50%). Correlation was considered statistically significant if the *p*-value was <0.05. Statistical analysis was performed using SPSS version 23.0.

For those primary cells which were sensitive to AS treatment, cell cycle phase distribution of cells were determined using flow cytometry (Yue et al., 2016). In addition, the expressions of cell proliferation (*cyclin D1*, *TOP2A*, and *RacGAP1*) and survival (*survivin*) genes in primary breast cancer cells were determined in eight AS-treated primary breast cancer cell samples using real-time PCR.

### Extraction of RNA, real-time PCR for Primary Breast Cancer Cells

Primary breast cancer cells were seeded in six-well culture plates and incubated for 24 h to allow attachment. AS extract (0.4–1.6 mg/ml) was then added into the wells and incubated for another 24 h. After treatment, cells were extracted using TRIzol Reagent (Thermo Fisher, USA) according to the manufacturer’s protocols. The RNA concentration was spectrophotometrically determined by a BioPhotometer (Eppendorf, USA). To quantify the amount of mRNA of *cyclin D1*, *TOP2A*, *RacGAP1*, *survivin*, and *GAPDH*, reverse transcription polymerase chain reaction (RT-PCR) was performed in Bio-Rad CFX96™ real-time system C1000 thermal cycler using the QuantiFast SYBR Green RT-PCR Kit from Qiagen (USA). Each 10-µl PCR reaction mix contained 100 ng RNA, 5 µl 2x QuantiFast SYBR Green RT-PCR Master Mix, 0.08 µl QuantiFast RT Mix, RNase-free water, and 1 µl of both the specific primers (20 µM); primer sequences were listed in **Table A1** **(in Appendix)**, which were synthesized by Life Technologies (NY, USA). Reactions were performed in triplicate using the following protocol: pre-incubation at 50°C for 10 min, then 95°C for 5 min followed by 40 PCR cycles at 95°C for 10 s and 60°C for 30 s. A melt curve analysis was performed at the end of the reaction to assess the specificity of the amplification. Relative quantification was obtained by the comparative threshold cycle (∆∆Ct) method (CFX Manager Software, version 1.6, Bio-Rad, Hong Kong). The specific gene mRNA levels were normalized to that of the internal control GAPDH and then expressed as the fold change compared to the control group. All samples were tested for one time.

### Orthotopic Breast Xenograft-Bearing Nude Mice Model

All animal studies in this study were approved by Animal Experimentation Ethics Committee of The Chinese University of Hong Kong (Ref. No. 14/066/MIS). The dosages of AS extract for mice were 0.75 (AS-L) and 1.5 (AS-H) g/kg, which are the lowest and highest human equivalent daily dose recommended in Chinese Pharmacopoeia 2015, respectively.

The *in vivo* effects of AS were determined in MDA-MB-231 tumor xenograft-bearing mice as MDA-MB-231 cell line was sensitive towards AS treatment, especially at low concentrations of AS. Female NOD/SCID mice (5–7 weeks of age) supplied by Laboratory Animal Services Centre, CUHK, were fed on a standard laboratory diet with free access to water. The mice were inoculated with MDA-MB-231 cells (5 × 10^6^ in 50 µl PBS plus 50 µl Matrigel) into the mammary fat pad to establish orthotopic xenografts. The mice were randomized into different groups (n = 13–16) when the tumors reached the volume of 70 mm^3^. AS extract (redissolved in water) was applied to the xenograft-bearing mice for 4 weeks. During the study, mice were kept in pathogen-free conditions with free access to food and water, monitored for impaired posture or movement. Body weights were monitored and the size of tumors was measured with a caliper twice a week and was calculated with the formula: length × (width)^2^/2 (Luo et al., 2014).

At the end of treatment period, the mice were anaesthetized by i.p. injection of ketamine and xylazine. The tumors were excised from the mice after cervical dislocation and weighed. Half of the tumors were fixed and processed for histological analysis. The cell proliferation (Ki-67 staining, anti-Ki67 from Abcam, UK), apoptosis ([Terminal deoxynucleotidyl transferase dUTP nick end labeling (TUNEL) staining kit, Roche, Switzerland], and blood vessels [CD31 and vascular endothelial growth factor (VEGF) staining, anti-CD31 from Dianova, USA, and anti-VEGF from Abcam, UK] were determined in tumor sections (Yue et al., 2016; Li et al., 2018).

### Orthotopic Syngeneic Breast Tumor-Bearing BALB/c Mice Model

The effects of AS were evaluated in orthotopic mouse breast tumor-bearing immune competent mouse models, so that the role of immunomodulation stimulated by the herbal medicines could be elucidated. Mouse tumor cells 4T1 (5 × 10^5^ in 50 µL PBS) were injected into mammary fat pads of female BALB/c mice of 6–8 weeks on day 1. The mice were randomized into different groups (n = 16–20) when the tumors reached the volume of 70 mm^3^. Mice were treated with AS extract (redissolved in water) for 4 weeks. In another set of experiments, mice with tumor (volume reached 70 mm^3^) were treated with cyclophosphamide (CTX, Sigma-Aldrich, USA) to induce blood deficiency syndrome (Li et al., 2011; Gu et al., 2013; Zhang et al., 2014). Mice were injected intraperitoneally with CTX (100 mg/kg) for three consecutive days. Then AS extract was applied to the CTX-treated tumor-bearing mice for 4 weeks. During the study, mice (n = 13–15 in each group) were kept in pathogen-free conditions with free access to food and water. Mice from each group were monitored for impaired posture or movement. Body weights were monitored and the size of tumors was measured with a caliper twice a week and calculated with the formula: length × (width)^2^/2.

At the end of treatment period, the mice were anaesthetized by i.p. injection of ketamine and xylazine and blood was collected by intra-cardiac puncture. The complete cell count and standard blood chemistry were measured. The spleens, lymph nodes, and tumors were excised from the mice after cervical dislocation and were weighed. Half of the tumors were fixed and processed for histological analysis. The cell proliferation (Ki-67 staining), apoptosis (TUNEL staining), tumor infiltrating lymphocytes (TILs, anti-CD8 from Roche, Switzerland) staining (Lechner et al., 2013), and blood vessels (CD31 staining) were determined in tumor sections. The excised spleens, lymph nodes, and another half of tumors were used to isolate lymphocytes. The lymphocytes were stained with fluorescence-conjugated anti-CD4 and anti-CD8 IgG for T-lymphocytes, anti-CD4, anti-CD25, and anti-Foxp3 for regulatory T cells, as well as anti-CD11b and anti-Gr1 for myeloid-derived suppressive cells by flow cytometry analysis. All the antibodies used in flow cytometry analysis were from BD Pharmingen, USA. The spleen lymphocytes were also cultured and activated with anti-CD3 (eBioscience, USA) monoclonal antibody. The cytokines such as IL-2, IL-12, IFN-γ, and TNF-α levels in the lymphocyte culture supernatants were measured using commercially available ELISA kits (BD Pharmingen, USA).

### Statistical Analysis

The differences among treatment and control groups were determined by one-way ANOVA, followed by *post hoc* Tukey’s multiple comparisons test or Dunnett test, or by Student’s t-test, depending on data distribution. All statistical analyses were performed using GraphPad PRISM software version 6.0 (GraphPad Software, USA). Data were expressed as mean ± standard error of mean (S.E.M.) for *in vivo* studies, and mean ± standard deviation (S.D.) for *in vitro* assays. In all comparisons, *p* < 0.05 was considered as statistically significant.

## Results

### Chemical Profile of Herbal Extract

The dried roots of *A. sinensis* (recorded as *Angelicae Sinensis* Radix in Chinese Pharmacopoeia) were purchased from suppliers in Hong Kong. The authenticated samples were subjected to quantification of ferulic acid (Chinese Pharmacopoeia recommended chemical markers) content. The AS herbal sample with 0.055% (w/w) ferulic acid, which was higher than the minimum amount of ferulic acid 0.05% (w/w) in dried raw herb according to Chinese Pharmacopoeia, was used for preparation of aqueous extract for the present study. Chemical marker ferulic acid and another active component ligustilide could be detected in the *A. sinensis* aqueous extract as shown in the UPLC chromatogram (**Figure 1B**). The percentage yield of *A. sinensis* aqueous extract was 65.4% (w/w) and the amounts of ferulic acid and ligustilide in the aqueous extract were 0.079% and 0.010% (w/w), respectively.

### AS Extract Exhibited Differential Effects on the Viability and Proliferation in Human Breast Cancer Cell Lines

To evaluate the *in vitro* proliferative activities of AS extract, four human breast cancer cell lines with different immunoprofiles, namely MDA-MB-361 (ER+HER2+), MCF-7 (ER+HER2-), SKBR3 (ER-HER2+), and MDA-MB-231 (ER-HER2-), were used. These four human breast cancer cell lines were treated with AS extract (0.4 to 6.4 mg/ml) for 48 h. Since estrogen is naturally found in women, ER-positive cells (MDA-MB-361 and MCF-7 cells) were also exposed to AS extract (0.4 to 6.4 mg/ml) in combination with 17β-estradiol (0.1 µM). The effects of AS extract on cell viability and proliferative responses were determined by MTT assay and BrdU cell proliferation assay, respectively, and the results of assays were expressed as mean fold of untreated control. The effects of AS extract on cell viability in ER-positive MDA-MB-361 and MCF-7 cells in the presence or absence of estradiol, as well as ER-negative MDA-MB-231 and SKBR3 cells, were shown in **Figure 1C**. AS extract (0.8 to 3.2 mg/ml) significantly increased cell viability in the presence of 17β-estradiol in MDA-MB-361 cells, with maximal 120% of the control, while the cell viability was also increased at 3.2 mg/ml (up to 121% of the control) when 17β-estradiol was absent. Besides, AS extract at 0.8 mg/ml increased cell viability in MDA-MB-231 cells to 113% of the control. The proliferation of MDA-MB-361 cells (with 17β-estradiol) and MDA-MB-231 cells were found to be stimulated by AS treatment, with the maximal 115% and 111% of the control, respectively (**Figure 1D**). For the MCF-7 and SKBR3 cell lines, the cell viability and proliferation were shown to be slightly decreased after AS treatments at 3.2 to 6.4 mg/ml. Thus, the *in vivo* effects of AS extract were later on determined in MDA-MB-231 xenograft-bearing mice as this cell line was sensitive towards AS treatment.

### AS Extract Could Increase Cell Viability and Proliferation in Human Primary Breast Cancer Cells?

**Figure 2:**
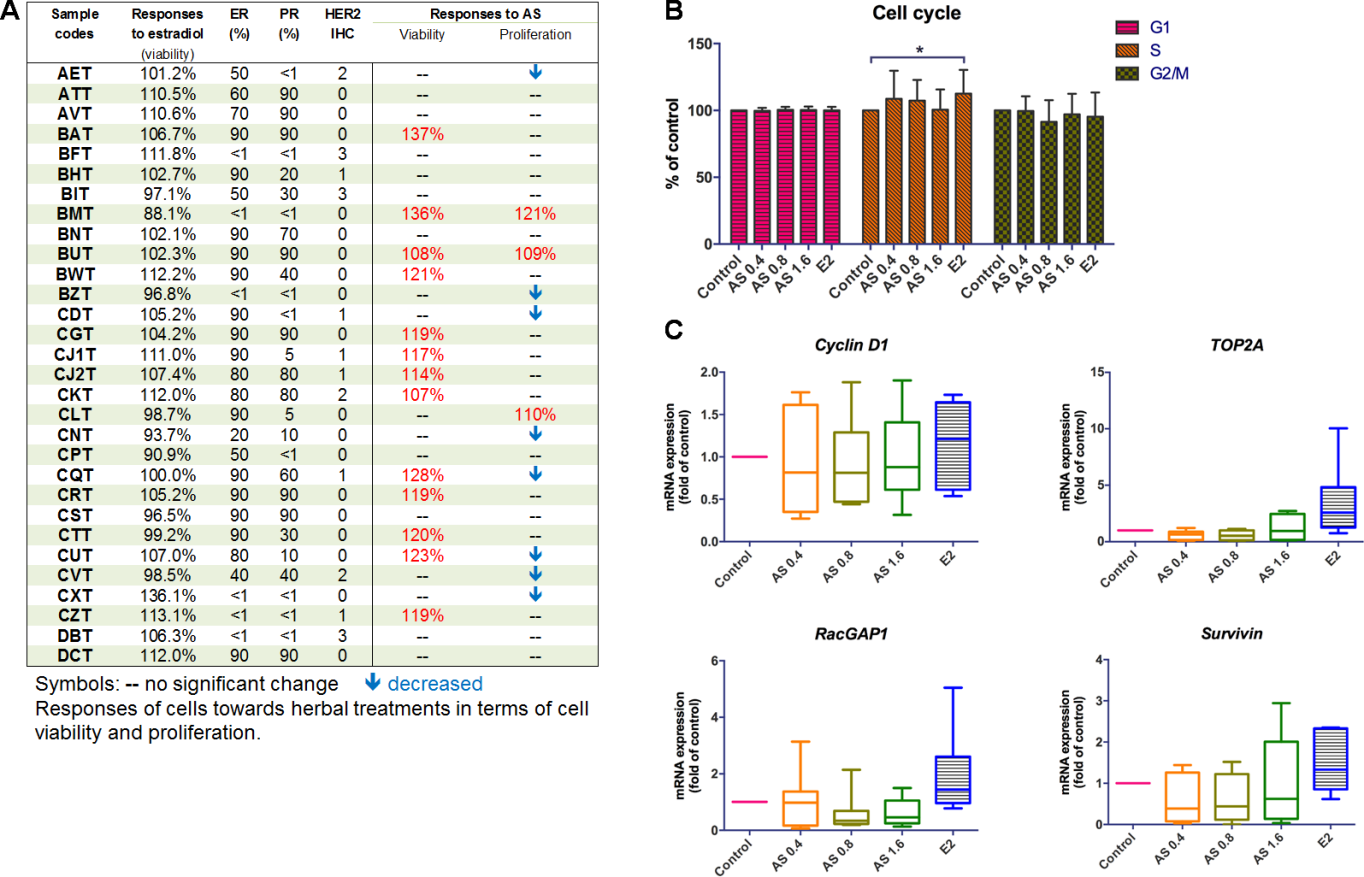
Effects of AS aqueous extract on human primary breast cancer cells. **(A)** Summary of ER, PR, HER2 expressions, and the responses of cells towards AS extract treatments (0.4, 0.8, or 1.6 mg/ml) or 17β-estradiol (E2, 0.1 µM) in epithelial cell-enriched primary breast cell samples. **(B)** Cell cycle phase distribution of 25 samples of human primary breast cancer cells. Data were expressed as mean percentage of control. **(C)** mRNA expressions of proliferation-related genes (n = 8). Data were normalized to corresponding GAPDH expressions in control cells and were expressed as mean fold of control. Mean + SD; statistical differences were determined by one-way ANOVA, followed by Dunnett test, with *p < 0.05, **p < 0.01, ***p < 0.001 against untreated control.

The potential stimulatory activities of AS extract were further verified in human primary breast cancer cells. Breast tumor tissues from consented breast cancer patients were obtained and used for breast cancer cells isolation. The epithelial cell-enriched primary cell samples were subjected to MTT viability and BrdU proliferation assays, respectively. The proliferative responses of the epithelial cell-enriched primary cell samples were diverse so the individual responses were shown (**Figure 2A**). Among 30 epithelial cell-enriched primary cell samples, 13 of them were shown to exhibit increased cell viability after AS extract (0.4–6.4 mg/ml) treatments. The cell proliferation in three cell samples (BMT, BUT, and CLT) were found to significantly increase after AS treatments. As the responses towards AS extract of different molecular subtypes of breast cancer cell lines have been evaluated and demonstrated in previous sections, attempts have been made to correlate the responses towards AS extract of primary breast cancer cells to their ER, PR, and HER2 status. As shown in **Figure 2A**, 18 of them exhibited relatively high ER percentages (≥ 70%), whereas the remaining samples had relatively low ER expressions. In epithelial cell-enriched samples, a trend of positive association was found with proliferative responses towards AS extract and ER expression (*p* = 0.071, **Table 1**).

**Table 1:** Among 30 samples, correlation of 17β-estradiol and AS extract response to clinico-pathological features (significant response and high epithelial contents).

A) 17β-estradiol					
		Correlation to 17β-estradiol			
		No	Yes	Total	*P*-value
Grade	1	3	0	3	0.734
	2	10	1	11	
	3	8	0	8	
	Total	21	1	22	
Tumor size	<2	14	1	15	1.00
	> = 2	8	0	8	
	Total	22	1	23	
Age	< = 55	11	1	12	0.622
	>55	14	4	18	
	Total	25	5	30	
Subtype	Lum A	6	1	7	0.478
	Lum B	15	2	17	
	HER2-OE	1	0	1	
	TNBC	3	2	5	
	Total	25	5	30	
ER	< = 70%	11	2	13	1.00
	>70%	14	3	17	
	Total	25	5	30	
PR	< = 70%	16	4	20	0.640
	>70%	9	1	10	
	Total	25	5	30	
HER2	Neg	22	5	27	1.00
	Pos	3	0	3	
	Total	25	5	30	
Ki67	<20%	7	1	8	1.00
	> = 20%	18	4	22	
	Total	25	5	30	
B) AS					
		Correlation to AS			
		No	Yes	Total	*P*-value
Grade	1	2	1	3	0.416
	2	5	6	11	
	3	6	2	8	
	Total	13	9	22	
Tumor size	<2	8	7	15	1.00
	> = 2	5	3	8	
	Total	13	10	23	
Age	< = 55	9	3	12	0.141
	>55	8	10	18	
	Total	17	13	30	
Subtype	Lum A	3	4	7	0.508
	Lum B	11	6	17	
	HER2-OE	1	0	1	
	TNBC	2	3	5	
	Total	17	13	30	
ER	< = 70%	10	3	13	0.071
	>70%	7	10	17	
	Total	17	13	30	
PR	< = 70%	13	7	20	0.255
	>70%	4	6	10	
	Total	17	13	30	
HER2	Neg	14	13	27	0.238
	Pos	3	0	3	
	Total	17	13	30	
Ki67	<20%	4	4	8	0.698
	> = 20%	13	9	22	
	Total	17	13	30	

### AS Extract Altered Cell Cycle Phase Distribution of Primary Human Breast Cancer Cells

The influence of AS extract on the cell cycle phase distribution of primary breast cancer cells were determined using flow cytometry. There were 25 epithelial cell-enriched primary cell samples treated with AS extract for 48 h and then subjected to flow cytometry analysis. The cell cycle phase distribution of primary cell samples after AS treatments was recorded. The percentages of each cell cycle phases compared to control were summarized in **Figure 2B**. Results showed that there was an increase in the numbers of cells undergoing S phase (AS extract at 0.4 and 0.8 mg/ml) while the numbers of cells undergoing G2 phase were decreased in a concentration-dependent manner. Besides, the significant increases of S phase in primary breast cancer cells were observed after 17β-estradiol (E2) treatment, confirming the primary breast cancer cells responses to such stimulatory activities.

To further elucidate the underlying mechanisms of potential stimulatory action of AS extract in primary breast cancer cells, the mRNA expressions of cell proliferation (*cyclin D1*, *TOP2A*, and *RacGAP1*) and survival (*survivin*) genes in eight epithelial cell-enriched primary breast cancer cell samples were determined using real-time PCR after AS (0.4–1.6 mg/mL) or 17β-estradiol (E2, 0.1 µM) treatments. The expressions of *cyclin D1*, *TOP2A*, and *RacGAP1* were found to be slightly up-regulated in the primary breast cancer cell samples treated with E2 (**Figure 2C**). In contrast, AS treatments did not induce apparent changes on the expressions of these proliferation-related genes.

### AS Treatment Did Not Stimulate the Growth of Orthotopic MDA-MB-231 Human Breast Xenografts

**Figure 3:**
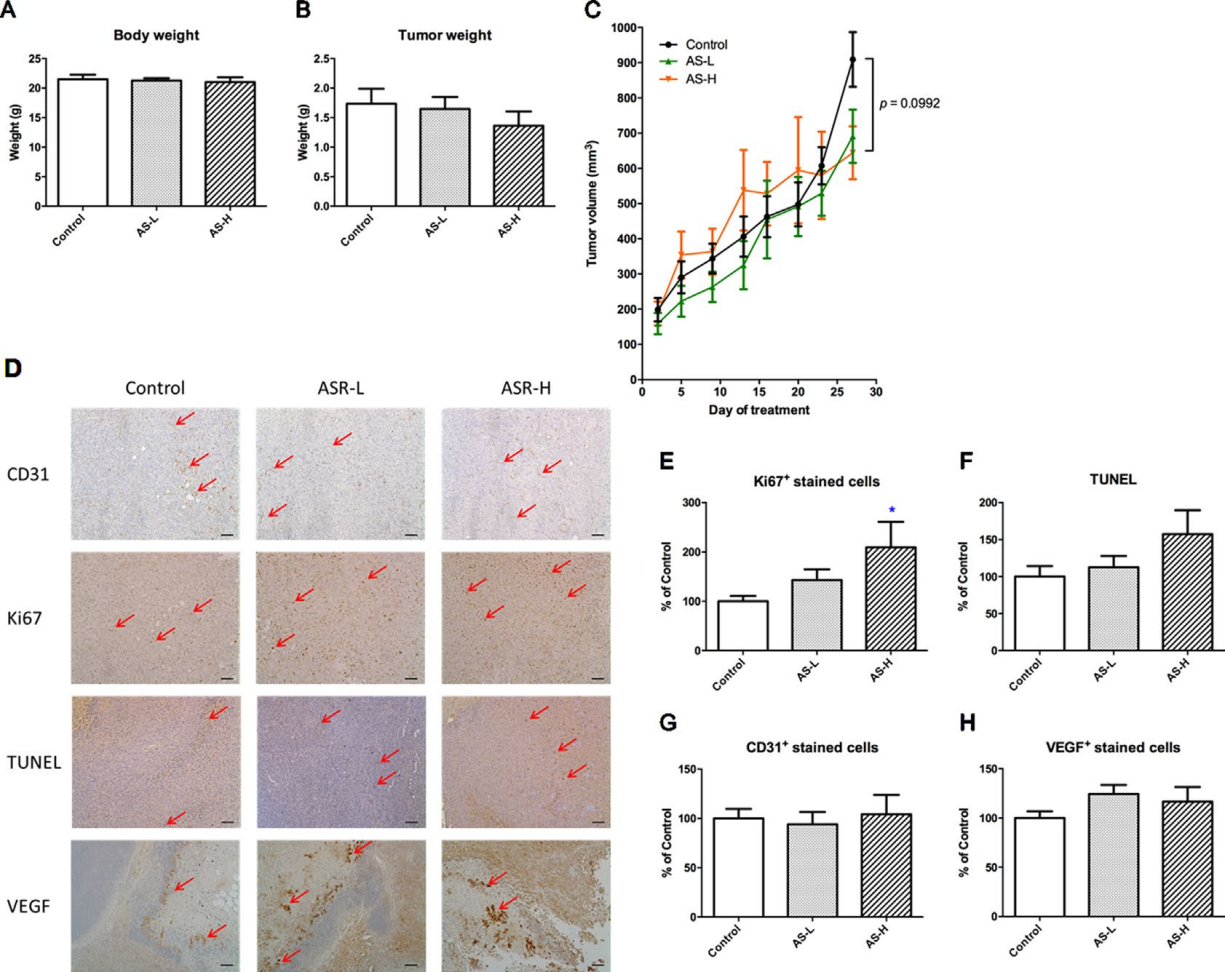
Tumor growth of MDA-MB-231 xenograft-bearing mice. Mice were treated with AS-L (0.75 g/kg) or AS-H (1.5 g/kg) daily for 4 weeks. **(A)** Body and **(B)** tumor weights of mice after AS treatments were recorded at the end of treatment. Data were expressed as mean + SEM, n = 13–16. **(C)** Tumor volume of mice in each group was measured twice a week during treatments. **(D)** Effects of AS treatments on the expressions of Ki67, CD31, VEGF, and the apoptotic area (assessed using TUNEL assay) in the tumor sections excised from MDA-MB-231 xenografts-bearing mice. Representative images of immunohistochemical staining in the tumor sections (magnification = 100×, scale bar: 100 µm). **(E–H)** Quantification of positive staining cells in tumor sections was conducted in a blinded manner. Data were expressed as mean + SEM, n = 13–16. Differences among treatment groups were determined by one-way ANOVA followed by *post hoc* Tukey’s multiple comparisons test. *p < 0.05, as compared with control group.

To examine the effects of the AS extract on tumor growth, MDA-MB-231 xenograft-bearing mice were treated with AS extract (p.o.) for 4 weeks. There was no significant body weight change observed in any treatment groups after 4 weeks of treatment (**Figure 3A**). As shown in **Figure 3B**, the final tumor weights were reduced 22% in AS extract high dose (AS-H, 1.5 g/kg) group when compared with untreated control group. From the tumor growth curves (**Figure 3C**), tumor volume of AS-H group was slightly higher than that of the control group from days 13 to 20, whereas such increases slowed down at the end of the experiment. Neither final tumor weight nor tumor volume on day 28 of AS-H group was significantly different from those of untreated control group (AS-H vs control, *p* = 0.0992).

The effects of AS treatments on the proliferation, apoptosis, and angiogenesis of xenografts were further investigated. Immunohistochemical staining with Ki67, CD31, and VEGF antibodies as well as TUNEL assay were performed in the tumor sections (**Figure 3D–H**). Using the semiquantitative scoring of immune-reactivity in tumor as shown in **Figure 3E**, the Ki67-positive stained cells in tumor sections were significantly increased in AS-H group when compared with untreated control group (*p* < 0.05). Meanwhile, the apoptotic cells determined by TUNEL assay were also shown to be increased in AS-H group, but the difference between treated and untreated groups was not statistically significant (**Figure 3F**). For the angiogenesis markers, AS treatments slightly increased VEGF-positive stained cells in tumors (**Figure 3H**), whereas they had no effect on the numbers of CD31-positive stained cells (**Figure 3G**). These results suggested that AS treatments affected the proliferation of cancer cells in tumors. At the same time, some of the components in AS might also regulate apoptosis in tumors, which resulted in only mild increases of tumor sizes after AS treatments as observed. There was no evidence supporting the promotive effects of 28-day AS treatment at the Chinese Pharmacopoeia recommended dosages (AS-L: 0.75 g/kg; AS-H: 1.5 g/kg) on tumor growth in MDA-MB-231 xenograft-bearing immunodeficient mice.

### Effects of AS Treatment on Orthotopic 4T1 Mouse Breast Tumors

**Figure 4:**
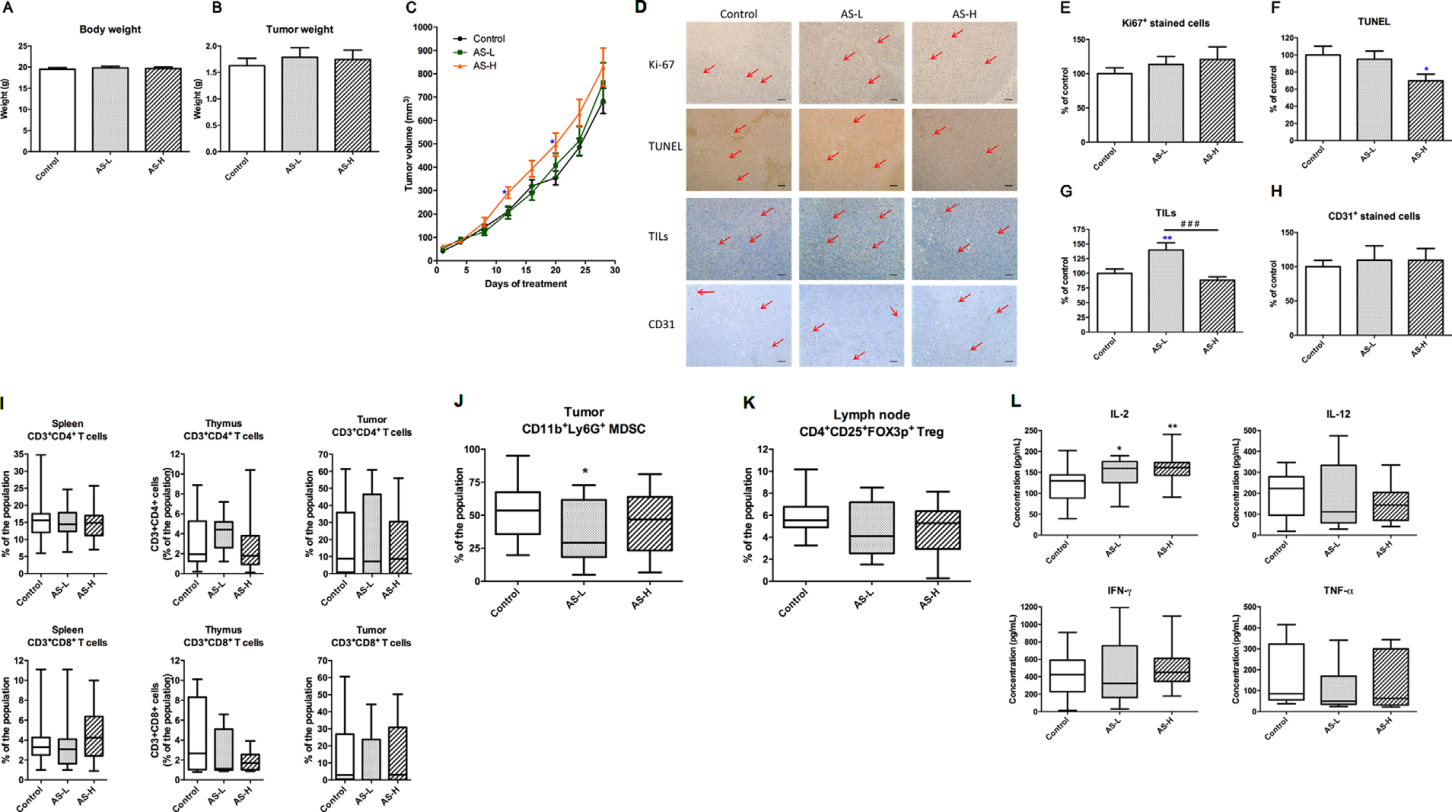
Tumor growth of 4T1 tumor-bearing mice. Mice were treated with AS-L (0.75 g/kg) or AS-H (1.5 g/kg) daily for 4 weeks. **(A)** Body and **(B)** tumor weights of mice after AS treatments were recorded at the end of treatment. Data were expressed as mean + SEM, n = 16–20. **(C)** Tumor volume of mice in each group was measured twice a week during treatments. **(D–H)** Effects of AS treatments on the expressions of Ki67 and CD31 in the tumor sections. The number of tumor infiltrating lymphocytes (TILs) and the apoptotic area (assessed using TUNEL assay) were also assessed in the tumor sections excised from 4T1 tumors. **(D)** Representative images of immunohistochemical staining in the tumor sections (magnification = 100×, scale bar: 100 µm). **(E–H)** Quantification of positive staining cells in tumor sections was conducted in a blinded manner. Data were expressed as mean + SEM, n = 16–20. Statistical differences were determined by one-way ANOVA, followed by *post hoc* Tukey’s multiple comparisons test. *p < 0.05, **p < 0.01, as compared with control group. ^###^p < 0.001 as compared among different groups. **(I–L)** The immune responses of 4T1 tumor-bearing mice. Lymphocytes were isolated from the mice treated with AS-L (0.75 g/kg) or AS-H (1.5 g/kg) daily for 4 weeks. Changes in the populations of **(I)** CD3+CD4+ (cytotoxic T-cells), CD3+CD8+ (helper T-cells) in spleen, thymus, and tumor, **(J)** myeloid-derived suppressor cells in tumor, and **(K)** regulatory T-cells in lymph node were determined using flow cytometry. **(L)** The levels of cytokines (IL-2, IL-12, IFN-γ, and TNF-α) of spleen lymphocytes (co-incubated with CD3). Data were expressed as mean + SEM, n = 9–16. Statistical differences were determined by one-way ANOVA, followed by *post hoc* Tukey’s multiple comparisons test. *p < 0.05, **p < 0.01 as compared with control group.

The orthotopic mouse breast tumor-bearing immune competent BALB/c mice model was then used to evaluate the effects of AS extract on tumor growth and microenvironment. Mice were inoculated with mouse breast 4T1 cancer cells into mammary fat pads and, after a week, were treated with AS extract (0.75 or 1.5 g/kg orally) daily for 4 weeks. After 4-week AS treatment, mice were sacrificed and the body and tumor weights were measured. The final body and tumor weights in AS treatment groups were not significantly changed (**Figure 4A and B**). Nevertheless, tumor volume in mice treated with AS-H (1.5 g/kg) was significantly higher than that in untreated control mice on days 13 and 20 (*p* < 0.05, **Figure 4C**). While the final tumor volume of AS-H group was not significantly different from the control group, suggesting the stimulatory effect of AS extract on 4T1 mouse tumor growth would be eliminated after prolonged treatments.

Tumor sections from each treatment groups were subjected to immunohistochemical staining with Ki67, CD31, and CD8 (for TILs) antibodies as well as TUNEL assay. As shown in **Figure 4D, E, and F**, in AS treatment groups, the numbers of Ki67 positive-stained cells were slightly increased, whereas the numbers of apoptotic cells were significantly decreased. These changes might partly contribute to the increased tumor volume. On the other hand, AS-L treatment sharply increased the number of TILs in tumors, whereas AS-H treatment did not (**Figure 4G**). Besides, the number of CD31 positive-stained cells has not been changed after AS treatments (**Figure 4H**).

The immune responses of immune competent tumor-bearing mice towards AS treatments were determined through lymphocyte subtypes characterization and cytokine productions. The CD4^+^ T cells and CD8^+^ T cells populations in spleen, thymus, and tumor have not been changed after AS treatments (**Figure 4I**). On the contrary, the populations of regulatory T cells (Treg, CD4^+^CD25^+^Foxp3^+^) in tumors and myeloid-derived suppressive cells (MDSC, CD11b^+^Gr1^+^) in lymph nodes were modulated after AS treatments (**Figure 4J and K**). For the *ex vivo* cytokine productions of spleen lymphocytes from different treatment groups, the production of IL-2 was significantly increased in AS-treated groups (**Figure 4L**). Summarizing the above data suggested that AS treatments would also exert anti-tumor activities via modulating systemic and local (within tumors) immune responses.

### Effects of AS Treatment on Orthotopic 4T1 Mouse Breast Tumors in the Presence of Cyclophosphamide

Since the potential “stimulatory” activities of AS treatments in breast cancer (in both human xenografts and mouse syngeneic tumors models) have been disproven, the activities of AS in breast tumor-bearing mice with chemotherapeutic, cyclophosphamide (CTX), were further investigated to provide more comprehensive evidences for the safety use of AS in breast cancer.

**Figure 5:**
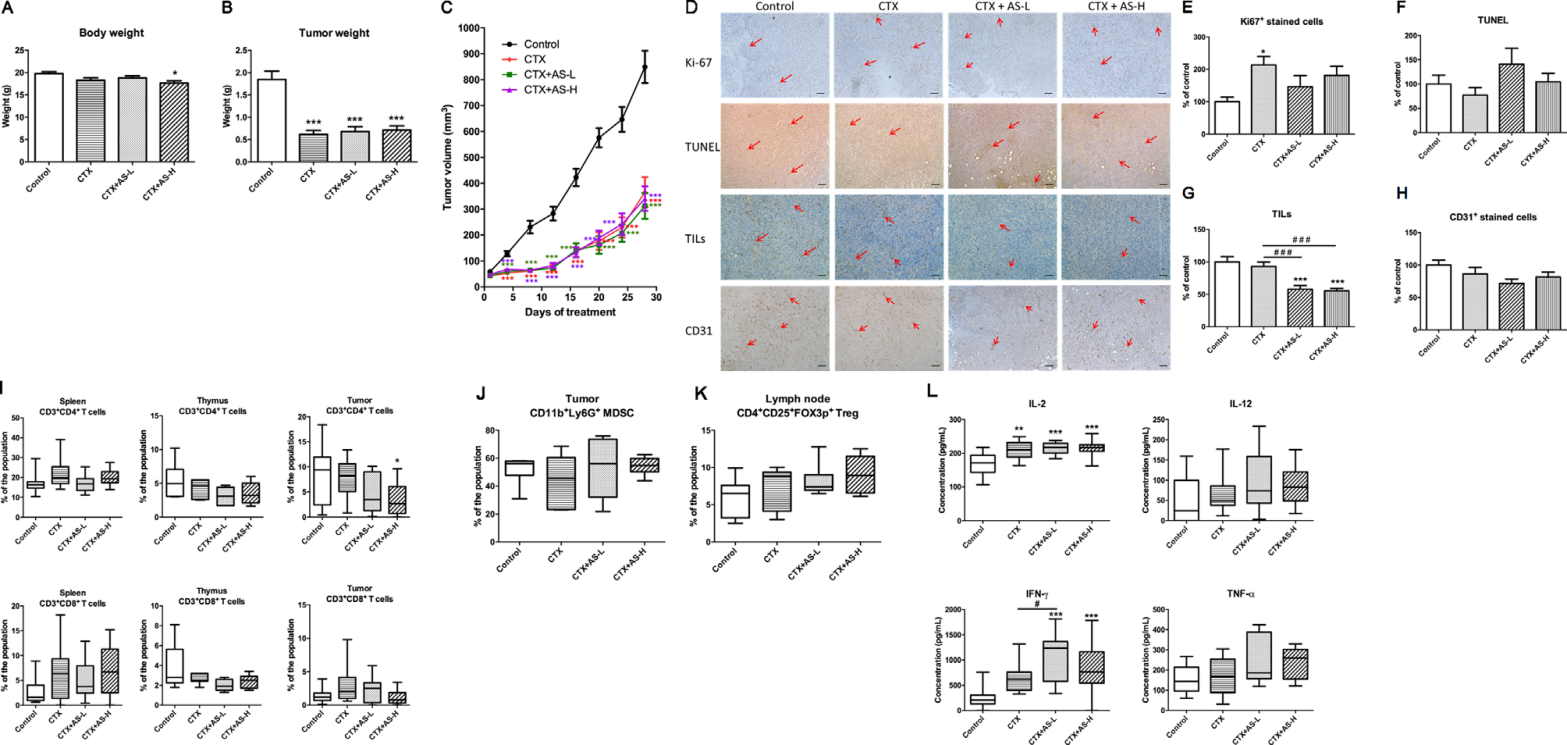
Tumor growth of 4T1 tumor-bearing mice treated with cyclophosphamide (CTX). Mice with tumors were treated with cyclophosphamide (CTX) for 3 days, then followed by treatment with AS-L (0.75 g/kg) or AS-H (1.5 g/kg) daily for 4 weeks. **(A)** Body weights and **(C)** tumor volume of mice in each group were measured once every 3 days during treatments. Data were expressed as mean ± SEM, n = 13–15. **(B)** Tumor weights of mice after AS treatments were recorded at the end of treatment. **(D)** Representative images of immunohistochemical staining in the tumor sections (magnification = 100×, scale bar: 100 µm). **(E–H)** Quantification of positive staining cells in tumor sections was conducted in a blinded manner. Data were expressed as mean + SEM, n = 13–15. Statistical differences were determined by one-way ANOVA, followed by *post hoc* Tukey’s multiple comparisons test. *p < 0.05, ***p < 0.001, as compared with control group. ^###^p < 0.001 as compared among different groups. **(I–L)** The immune responses of 4T1 tumor-bearing mice treated with CTX. Changes in the populations of **(I)** CD3+CD4+ (cytotoxic T-cells), CD3+CD8+ (Helper T-cells) in spleen, thymus, and tumor, **(J)** myeloid-derived suppressor cells in tumor, and **(K)** regulatory T-cells in lymph node were determined using flow cytometry. **(L)** The levels of cytokines (IL-2, IL-12, IFN-γ, and TNF-α) of spleen lymphocytes (co-incubated with CD3). Data were expressed as mean + SEM, n = 13–15. Statistical differences were determined by one-way ANOVA, followed by *post hoc* Tukey’s multiple comparisons test. *p < 0.05, **p < 0.01, ***p < 0.001, as compared with control group. ^#^p < 0.05 as compared among different groups.

As mentioned, according to Chinese medicine theory, AS is usually prescribed for cancer patients with blood-deficiency syndrome, especially after chemotherapy. Hence, the immunological and hematological parameters were determined in the breast tumor-bearing mice after CTX and/or AS treatments. Mice were inoculated with mouse breast 4T1 cancer cells into mammary fat pads and, after a week, were treated with CTX (100 mg/kg for three consecutive days) and then followed by AS extract (0.75 or 1.5 g/kg) orally daily for 4 weeks. After 4-week AS treatment, mice were sacrificed and the body and tumor weights were recorded. From the results, mice in all CTX-treated groups were decreased in body weights (**Figure 5A**). From **Figure 5B and C**, the tumor growth was apparently inhibited by CTX treatments and the final tumor weights were significantly reduced in all CTX-treated groups when compared with untreated control group. There was no significant difference among CTX+AS-treated and CTX-alone groups.

The cell proliferation and apoptosis in tumor sections were also determined in order to assess the effects of AS treatments in tumor-bearing mice. As shown in **Figure 5D and E**, the number of Ki67 positive-stained cells in tumor sections was found to be significantly higher in CTX-alone treatment group when compared with untreated control group. Subsequent AS treatments did not significantly reverse the increases but with the trend of decrease. In addition, the signal of apoptotic cells (shown in TUNEL staining) in CTX+AS-L treatment group slightly increased among all treatment groups (**Figure 5F**). Interestingly, the TILs and CD31 positive-stained cells were decreased in CTX plus AS treatment groups (**Figure 5G and H**).

On the other hand, complete cell count and standard blood chemistry were measured in mice treated with or without CTX and/or AS-H to evaluate the side effects of the treatments. As shown in **Table 2**, the severe reduction of WBC count, neutrophils, monocytes, eosinophils, and basophils could be observed in CTX treatment groups. The counts of these cells were increased in different extents after AS-H treatment for 4 weeks, although there was no significant difference between CTX and CTX+AS-H groups. In contrast, the inhibitory effects of CTX on the RBC count and hemoglobin concentration (Zhang et al., 2014) were eliminated, which might be due to the measurement being done 4 weeks after the exposure. As a result, the additional AS-H treatment did not significantly affect these parameters.

**Table 2:** Hematological parameters in non-tumor-bearing (naive) mice and 4T1 breast tumor-bearing mice after CTX and/or AS treatments.

	Naive	Control	CTX	CTX+AS-H
WBC count (k/µl)	4.03 ± 1.17^^^	225.00 ± 24.56	73.06 ± 9.89***	154.66 ± 28.11
Neutrophils (k/µl)	0.93 ± 0.23^^	199.24 ± 25.98	62.88 ± 9.12*	142.46 ± 27.28
Lymphocytes (k/µl)	2.87 ± 0.93^	18.74 ± 5.37	7.50 ± 1.07	7.92 ± 0.84
Monocytes (k/µl)	0.03 ± 0.03^^^	1.06 ± 0.13	0.42 ± 0.05***	0.68 ± 0.10*
Eosinophils (k/µl)	0.07 ± 0.03^^	3.44 ± 0.65	1.22 ± 0.37*	1.56 ± 0.35*
Basophils (k/µl)	0.00 ± 0.00^^	3.62 ± 0.99	0.52 ± 0.15*	1.36 ± 0.43
RBC count (M/µl)	9.71 ± 0.21	8.94 ± 0.19	9.48 ± 0.42	9.43 ± 0.25
Hemoglobin (g/dl)	14.87 ± 0.18	13.56 ± 0.42	12.48 ± 0.55	13.38 ± 0.43
Hematocrit (%)	47.27 ± 2.28	42.82 ± 1.05	41.50 ± 1.50	41.68 ± 1.08
MCV (fl)	48.67 ± 1.74	46.42 ± 0.60	45.24 ± 0.52	44.18 ± 0.36
MCH (pg)w	15.33 ± 0.15^	14.50 ± 0.19	14.34 ± 0.16	14.16 ± 0.14
MCHC (g/dl)	31.60 ± 1.37	31.24 ± 0.38	31.68 ± 0.39	32.04 ± 0.20
RDW (%)	13.13 ± 0.19	13.54 ± 0.21	14.64 ± 0.29*	14.22 ± 0.18
Platelet count (k/µl)	693.33 ± 222.12	930.40 ± 40.61	804.40 ± 138.52	813.20 ± 41.47
MPV (fl)	10.23 ± 1.59	9.76 ± 0.16	9.96 ± 0.91	9.52 ± 0.38

Results were expressed as mean ± S.E.M. of five mice each group.

^p < 0.05, ^^p < 0.01, ^^^ p < 0.001, as compared between naive and control groups; *p < 0.05, **p < 0.01, ***p < 0.001, as compared between CTX vs control or CTX+AS-H vs control groups by one-way ANOVA followed by post hoc Tukey’s multiple comparison test.

CTX, cyclophosphamide (100 mg/kg for three consecutive days); AS-H, AS extract (1.5 g/kg orally daily for 4 weeks).

Furthermore, the immune responses of syngeneic tumor-bearing mice towards CTX+AS treatments were also determined through lymphocyte subtypes characterization and cytokines productions. The CD4^+^ T cells and CD8^+^ T cells populations in spleen, thymus, and tumor were reduced in CTX-treated groups while AS treatments did not resume the suppressed T cell populations (**Figure 5I**). Similarly, the altered populations of regulatory T cells (Treg, CD4^+^CD25^+^Foxp3^+^) in tumors and myeloid-derived suppressive cells (MDSC, CD11b^+^Gr1^+^) in lymph nodes were not changed after subsequent AS treatments (**Figure 5J and K**). Nevertheless, the *ex vivo* cytokines (IL-2, IFN-γ, and TNF-α) productions of spleen lymphocytes isolated from AS-treated mice were shown to be higher than those from CTX alone treatment (**Figure 5L**). These data suggested that AS treatments might still play a role in modulating immune response in tumor-bearing condition. However, the modulatory effect within the tumor (tumor microenvironment) was not noticeable as the tumor growth has already been controlled by CTX treatment.

## Discussion

A systematic pre-clinical research platform has been established for CHM safety evaluation in cancer management. It would serve as a new approach of safety/toxicology studies for herbal medicines, which are certainly infeasible and unethical to carry out in cancer patients. Here, the popular myth regarding the stimulatory activities of Donggui (*A. sinensis*, AS) in breast cancer has been disproved by the scientific evidences generated from the present study. Three levels of experiments, i.e., i) *in vitro* screening of AS in breast cancer cell lines with different molecular subtypes; ii) *in vitro* verification of the proliferative properties of AS extract in human primary breast cancer cells; and iii) *in vivo* systemic impact evaluation of AS extract in human breast xenograft-bearing mice and in syngeneic breast tumor-bearing mice, have been conducted.

Nowadays in Chinese populations worldwide, CHM consumption by cancer patients as adjuvant therapy is a common scenario (Lai et al., 2012; Fan et al., 2014; Wong et al., 2014). In general practice, Western medical providers and even some Chinese medicine practitioners will try to avoid using estrogenic herbs (such as AS) in breast cancer patients. Previous *in vitro* studies revealed the stimulatory activities of AS extracts (aqueous or organic solvent extract) in breast cancer cells (Amato et al., 2002; Lau et al., 2005; Zhu et al., 2007), while the effects of AS on breast cancer patients have seldom been scientifically evaluated in animal models. Furthermore, with the advancement of breast cancer classification, several key receptors of breast cancer cells, which are the therapeutic targets, were now identified. These receptors will play roles on the proliferative responses of cancer cells towards exogenous chemicals including CHM components, and eventually the growth of tumors will be altered. In this regard, four human breast cancer cell lines with different expressions of ER, PR, and/or HER2 have been used to verify if AS could be stimulatory to all of these cell lines.

Our results showed that AS extract did stimulate the proliferation of homogeneous breast cancer cell lines, especially the MDA-MB-231, MCF-7, and MDA-MB-361 cells. The increased levels of breast cancer cell viability were not as large as those in our previous study (Lau et al., 2005) (∼140–150% vs ∼120% in the present study). The discrepancy would be due to the different passages of breast cancer cell line, culture media, as well as the sources of AS herbal materials. Nonetheless, significant changes could be observed against untreated control. As both cell viability (reflected by MTT assay) and proliferation (determined by BrdU assay) of MDA-MB-231 cells were found to be significantly increased, the proliferative responses of these cells towards AS extract were then assessed in xenograft models.

Another breakthrough of the present study was the establishment of screening platform involving primary breast tissues from patients. In view of the unfeasibility to conduct clinical trial for CHM safety evaluation in breast cancer patients, therefore, primary breast cancer cells isolated from consented patients’ tissues were used to evaluate the potentially unsafe effects (proliferation of cancer cells) of CHM in a pre-clinical approach. The outcomes from this part of study were very valuable because the information, such as pathologic factors, biomarker expression, cancer subtypes, as well as the individual responses towards AS extract, etc. could be generated and analyzed as a whole. In comparison with previous *in vitro* evaluation using cancer cell lines only, the clinical-relevant data can reflect the actual circumstances in breast cancer management. Our results showed that a trend of positive association was found with proliferative responses towards AS and ER expression. Although the correlation did not reach statistical significance (p value was 0.071), the information is clear that AS treatment would have potential to stimulate the proliferation of ER-positive breast cancer cells. There will be plenty of prospects to explore the responses towards AS and other estrogenic CHM with other biomarkers/receptors by using this screening platform. In addition, there is limited information on molecular mechanisms as to how AS extract promotes or inhibits the proliferation of primary breast cancer cells. Our data from cell cycle analysis and real-time PCR suggested that AS extract exerted mild stimulatory activities in breast cancer cells at S phase; however, it did not up-regulate the tested proliferation-related genes, TOP2A and RacGAP1, as well as survival-related gene, survivin. On the other hand, although the cell viability and proliferation were found to be increased in ER-negative cell line (MDA-MB-231), the xenograft growth has not been stimulated after AS treatment in mice. Furthermore, among tested primary breast cancer cell samples, only two samples (BMT and CZT, ER expression <1%) were found to have increased cell viability. Taken together, there is not enough evidence at this stage to conclude whether AS extract would stimulate ER-negative breast cancer cells; hence, future investigations using primary breast cancer cells (with larger sample number) are warranted.

Despite the *in vitro* results, the 28-day oral treatment with AS extract did not result in increasing tumor growth in MDA-MB-231 xenograft-bearing mice; in contrast, there was a trend of reducing final tumor weights (**Figure 3**). From the immunostaining of tumor sections, AS extract at Chinese Pharmacopoeia-recommended high dose (1.5 g/kg, AS-H) was shown to enhance the proliferation of tumor cells, whereas AS treatment also increased the TUNEL signal in tumors. It is possible that different components in AS extract exhibited various actions on the tumors. However, the overall outcome was shown not to be stimulatory on MDA-MB-231 tumor growth. On the other hand, same AS treatment has been verified in the MDA-MB-361 (ER-positive and HER2-positive) xenografts in NOD/SCID mice. Similar results were obtained that the final weights of MDA-MB-361 xenografts were also reduced in AS-H treated group when compared with untreated control group (data not shown). These results implied that AS extract at the tested dosages would not stimulate the growth of tumor independent of the subtypes of ER/HER2.

Furthermore, the AS treatment was testified in the syngeneic tumor-bearing mice, in which the immune system was competent and the microenvironment was modulated by the immune cells.

Interestingly, significant augmentation of tumor growth was observed on days 13 to 20 in the AS-H treated group. Although there was no significant difference of final tumor weight among AS treatment groups and untreated control group, the increased proliferative tumor cells (shown by Ki67 staining) as well as reduced apoptosis in tumors (shown by TUNEL signal) were observed in the AS-H treatment group. The potential unfavorable signs, i.e., promoting tumor growth, of AS-H treatments were observed. Nonetheless, the changes of TILs and MDSC numbers in tumors as well as the Treg cells in lymph node might take part in counteracting the stimulated tumor growth. As a result, the stimulatory activities in AS-treated groups became mild after prolonged treatment to 28 days. Moderate regulation of tumor microenvironment by AS extract would account for the final outcomes.

In fact, cancer patients after chemotherapy will usually be prescribed with tonifying and/or invigorating herbs by Chinese medicine practitioners. Being one type of tonic/blood-replenish herbs, AS extract was verified for its activities in tumor-bearing mice with blood deficiency syndrome. Results showed that after CTX treatment, 28-day AS treatment did not affect the tumor growth and the final tumor weights, which have already been suppressed by CTX treatment. Meanwhile, the numbers of TILs were reduced and the IFN-γ production by spleen lymphocytes was significantly increased after AS treatment when compared to CTX-alone treatment. Since the anti-tumor effect of CTX was potent, it might have masked the effects of AS treatment on tumor growth. On the other hand, recent reports suggested that *A. sinensis* polysaccharide (ASP) could protect bone marrow stromal cells from oxidative injuries caused by 5-fluorouracil (Xiao et al., 2017) and AS also promotes hematopoietic activities, which potentially contribute to the blood enrichment effects of AS against APH- and CTX-induced BD mice (Hua et al., 2017). Some of the AS metabolites have also been identified to possess enriching-blood effect (Ji et al., 2018). However, the blood deficiency syndrome in the mice was not reflected by the blood chemistry profile in the present study. The long (25 days) recovery period for mice after CTX treatments may explain this observation. Besides, the AS-H dosage, which was recommended by the Chinese Pharmacopoeia, may not be high enough to exert the blood-replenish effect. Thus, the white blood cells replenishment rather than the blood-replenish effect of AS in this tumor-bearing model could be seen. Nonetheless, this is the clinical setting that breast cancer patients consume CHM after chemotherapy. The present data demonstrated the minor effect of AS in the CTX-treated mice, which may not be regarded as harmful to the patients who undergo chemotherapy.

From the data of tumor-bearing mice models, which are novel in anti-cancer CHM safety evaluation research, the queries or concerns of Chinese medicine practitioners on using AS in breast cancer patients could be partly answered. These data were not generated only from a single homogeneous cell line or an animal model, but from cell lines with different molecular subtypes and xenograft models with various microenvironment conditions (Killion et al., 1999; Kwon, 2014), which would provide more comprehensive aspects for herb safety screening.

Last but not least, the present study further confirmed that cell-based assay results would not be absolutely relevant to the *in vivo* results. The message of taking absorption/metabolism of herbal extracts into account for evaluating pharmacological efficacies would be another highlight of the present study. In contrast to the *in vitro* studies, which could well elucidate the underlying molecular mechanisms induced by AS extract/active compounds (Zhou et al., 2015; Ma et al., 2017; Qi et al., 2017), xenograft/tumor-bearing mice models (with or without chemotherapeutics) would have advantages to show the systemic influences/efficacies of the absorbed components of AS. Pharmacokinetics studies showed that the bioactive constituents, ferulic acid (Li et al., 2012), and ligustilide (our unpublished data) could be absorbed well in murine after orally taking AS extract. Recent metabolomics studies of AS also demonstrated that several metabolic pathways were involved in the pathological mechanisms of blood deficiency syndrome and the enriching-blood effect mechanism of AS, and the tested AS extract was orally administered to mice for a period of time (Hua et al., 2017; Ji et al., 2018). The importance of evaluating the efficacy after oral route of administration of herbal extracts, which is the most common practice for taking CHMs, is unquestionable.

In conclusion, our results showed that AS extract did stimulate the proliferation of homogeneous breast cancer cell lines in different extents. Both estrogen positive and negative breast cancer cells were sensitive to AS extract *in vitro* but in lesser extent when comparing with previous studies (Amato et al., 2002; Lau et al., 2005). By using the primary breast cancer cells, mild stimulating activities of AS extract were observed in individual cases. The correlation analysis revealed that the proliferative responses to AS extract associated with the expression of ER in the primary breast cancer cells. Our animal experiments also did not show significant stimulatory activities of AS on tumor growth after 28-day treatments in human breast xenograft- or syngeneic mouse breast tumor-bearing mice. However, a transient increase of tumor size in AS extract high dose treatment group during days 13 to 20 of treatments in 4T1 tumor-bearing mice should not be overlooked. Taking together, the present findings provided further scientific evidences, which showed that the estrogenic herb AS are not that stimulatory in breast cancer both *in vitro* and *in vivo*, though it should still be used with caution particularly in ER-positive breast cancer patients. This is the first systematic pre-clinical study to illustrate the safety use of AS in breast cancer patients by providing varied levels of evidences. The paradoxical perception of AS in promoting breast cancer growth could be dispelled, and these findings will affect future clinical practice. Furthermore, the new approach of applying breast cancer cell lines, xenograft, and syngeneic tumor models as well as primary breast cancer cells from patients’ tumors in CHMs safety evaluation was proven feasible. The systematic study approach described here would certainly provide a new way to verify whether the use of other estrogenic Chinese herbs in breast cancer is appropriate.

## Data Availability Statement

The data that support the findings of this study are available from the corresponding author upon reasonable request. The authors declare that data supporting the findings of this study are available within the paper and its supplementary information files.

## Ethics Statement

The study protocol of using human tissues was approved by the Joint Chinese University of Hong Kong–New Territories East Cluster Clinical Research Ethics Committee (CREC Ref. No.: 2014.336) and Union Hospital Ethics Committee (EC Ref. No. EC013). 

Guidelines for the care and use of animals from the Animal Experimentation Ethics Committee of the Chinese University of Hong Kong were followed. The protocol of the present study was approved by the Animal Experimentation Ethics Committee of the Chinese University of Hong Kong (Ref. No. 14/066/MIS).

## Author Contributions

CBL and GGY conceived the study. GGY, L-SW, H-WL, and SG performed the *in vitro* and *in vivo* studies and analyzed the data. JYT performed the characterization of primary breast cancer cells. BKL and GMT provided breast cancer patients’ tissue samples. Z-XL provided advices on Chinese medicines. CBL contributed essential reagents and tools. GGY and CBL wrote the manuscript. All authors reviewed the manuscript.

## Funding

This study was financially supported by Food and Health Bureau HKSAR, Health and Medical Research Fund no. 12130471.

## Conflict of Interest Statement

The authors declare that the research was conducted in the absence of any commercial or financial relationships that could be construed as a potential conflict of interest.

## Abbreviations

AS, *Angelica sinensis*; BrdU, bromodeoxyuridine; CAM, complementary and alternative medicine; CHM, Chinese herbal medicines; CTX, cyclophosphamide; E2, 17β-estradiol; ER, estrogen receptor; HER2, human epidermal growth factor receptor 2; MDSC, myeloid-derived suppressive cells; MTT, 3-(4,5-dimethyl-2-thiazolyl)-2,5-diphenyl-2H-tetrazolium bromide; PR, progesterone receptor; TILs, tumor infiltrating lymphocytes.

## Appendix

**Table A1:** Primers sequences of genes expressing in primary breast cancer cells.

Primer names	Sequence (5’ to 3’)

Cyclin D1 forward	CGCCCTCGGTGTCCTACTTCAA
Cyclin D1 reverse	GTGGCGACGATCTTCCGCAT
Survivin forward	CTGTGGGCCCCTTAGCAAT
Survivin reverse	TAAGCCCGGGAATCAAAACA
TOP2A forward	TGGCTGTGGTATTGTAGAAAGC
TOP2A reverse	TTGGCATCATCGAGTTTGGGA
RacGAP1 forward	GAATGTGCGGAATCTGTTTGAG
RacGAP1 reverse	TCGCCAACTGGATAAATTGGA
GAPDH forward	cgagatccctccaaaatcaa
GAPDH reverse	ggtgctaagcagttggtggt
